# Ion Flux Dependent and Independent Functions of Ion Channels in the Vertebrate Heart: Lessons Learned from Zebrafish

**DOI:** 10.1155/2012/462161

**Published:** 2012-11-13

**Authors:** Mirjam Keßler, Steffen Just, Wolfgang Rottbauer

**Affiliations:** Department of Internal Medicine II, University of Ulm, 89081 Ulm, Germany

## Abstract

Ion channels orchestrate directed flux of ions through membranes and are essential for a wide range of physiological processes including depolarization and repolarization of biomechanical activity of cells. Besides their electrophysiological functions in the heart, recent findings have demonstrated that ion channels also feature ion flux independent functions during heart development and morphogenesis. The zebrafish is a well-established animal model to decipher the genetics of cardiovascular development and disease of vertebrates. In large scale forward genetics screens, hundreds of mutant lines have been isolated with defects in cardiovascular structure and function. Detailed phenotyping of these lines and identification of the causative genetic defects revealed new insights into ion flux dependent and independent functions of various cardiac ion channels.

## 1. Introduction

Ion channels are pore-forming proteins that warrant controlled and directed flux of ions through membranes. Temporal and spatial coordination of ion movements is essential for a wide range of physiological processes including the generation and propagation of the membrane action potential that is critical for the biomechanical activity of muscle cells. Despite their well-established canonical electrophysiological functions in the heart, recent findings have demonstrated that ion channels also might feature ion flux independent functions during heart development and morphogenesis long before acting as ion-conducting pores. For example, targeted knockout of the cardiac sodium channel SCN5A in mice leads to embryonic lethality due to defective cardiogenesis. Besides the expected electrophysiological alterations, hearts of SCN5a-deficient mice develop a common hypoplastic ventricular chamber with reduced trabeculae whereas the endocardial cushions of the atrio-ventricular (AV) canal or the truncus arteriosus form normally [1]. These findings implicate that regular ion channel function is crucial not only for the regulation of heart rhythm but also for cardiogenesis.

Whereas the study of heart development in mice is hindered by the *in utero* development of the embryo and the high mortality of embryos with cardiac defects, animal models such as the zebrafish (*Danio rerio*) emerged as a powerful model organism to study cardiac development and function in the last few years.

The advantages of this animal model are external fertilization and fast development. The embryos can easily be reared in aqueous medium and are accessible for manipulation at all developmental stages. Meanwhile their organogenesis can be observed continuously due to their optical transparency [2]. During its embryonic development the zebrafish does not entirely depend upon a functional cardiovascular system and circulation, since oxygen is distributed by passive diffusion [3]. This facilitates extended studies on severe congenital cardiovascular defects. Besides, the almost completely sequenced genome of the zebrafish and the highly conserved gene functions compared to humans further strengthen the zebrafish's role for cardiovascular research [2, 4]. Large scale forward genetic screens in zebrafish expanded our knowledge on genetic networks that mediate development and maintenance of cardiac form and function. Genes identified in these screens encode for a wide variety of different proteins: transcription factors, signaling molecules, as well as several structural proteins of cardiomyocytes including the contractile apparatus.

**Figure 1:**
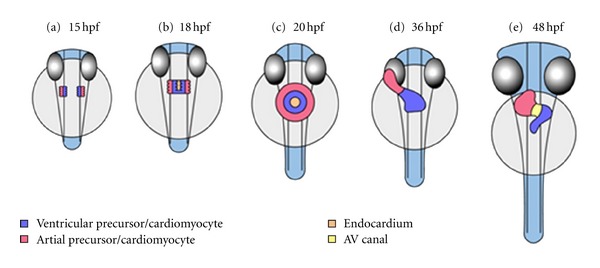
Stages of heart development in zebrafish embryos in a ventral view. (a) At 15 hpf cardiac precursors move towards the anterior lateral plate mesoderm; the atrial precursors are located laterally, the ventricular precursors medially. (b) Cardiac fusion starts at about 18 hpf at the posterior end of the bilateral heart fields; first, endocardial cells arrive at midline, followed by ventricular cells. The atrial precursors do so slightly later. (c) After cardiac fusion the cells form the cardiac cone. Viewed ventrally, this structure resembles a ring: endocardial cells lining the central lumen, and ventricular cells are surrounded by atrial precursors. The cardiac cone starts to transform into the heart tube by 21 hpf. (d) Cardiac looping of the heart tube occurs between 26 hpf and 48 hpf. The linear heart tube bends and creates an S-shaped loop. By 36 hpf the atrium and ventricle become distinguishable. (e) The heart tube rotates. The different parts of the heart tube do not rotate equally and therefore torsion occurs. The AV canal develops by constriction of the boundary between atrium and ventricle. Ventricular maturation by appositional growth takes place.

The key steps of heart development in zebrafish resemble those in humans and other mammals. Differentiation of cardiac precursors is required to form a heart tube that finally loops and builds an atrial and ventricular chamber separated by an atrio-ventricular (AV) valve. At 5 hours after fertilization (hpf) cardiac progenitor cells are located bilaterally in the lateral marginal zone. During gastrulation cardiac progenitor cells migrate towards the embryonic midline to end up at the anterior lateral plate mesoderm (Figure 1(a)). The cells continue to converge at midline at about 18 hpf (*cardiac fusion*; Figure 1(b)) and form the cardiac cone (Figure 1(c)). The shallow cone shifts from midline towards the left side (*cardiac jogging*). By 21 hpf a linear heart tube forms and exhibits regular peristaltic contractions. Endocardial cells lie within the inner lining of the lumen, myocardial cells peripherally. The heart tube elongates (*cardiac extension*) and bends to the right to create an S-shaped form (*cardiac looping,* Figure 1(d)). The future ventricle and atrium become distinguishable and, by 36 hpf, show sequential contractions with a characteristic AV delay. The AV canal becomes detectable and by 48 hpf the endocardial cells lining this canal form the endocardial cushions (Figure 1(e)). Gradually, between 48 hpf and 72 hpf, ventricular maturation proceeds, the ventricular wall thickens by appositional growth (*cardiac ballooning*), and trabeculae are formed. Endocardial cushions enlarge and, by 105 hpf, have differentiated into valve leaflets of the AV valve.

Heart morphogenesis is accompanied by cardiomyocyte specification and differentiation. Myocardial cell precursors start to express cardiac myosin genes including myosin light chain polypeptide 7 (myl7, cmlc2). At 16 hpf the medial cells differentiate into ventricular cells expressing ventricle myosin heavy chain (vmhc) and at 22 hpf the lateral, atrial myosin heavy chain (amhc) positive cells differentiate into atrial cells. The transcription factor nkx2.5 is needed to generate myocardial and endocardial progenitor cells, which is regulated by bone morphogenetic protein (Bmp), gata5, and hand2 (for reviews see [5–8]).

The role of ion channels in adulthood for the generation and the propagation of action potentials in excitable cells and the contraction of myocytes and cardiomyocytes is well understood [9]. Additionally, recent findings mainly from the zebrafish model demonstrate that ion channels also play a crucial and unexpected role in heart development. The presence of ion channels is required for normal cardiogenesis. A knockout or Morpholino antisense oligonucleotide mediated knockdown of ion channel genes in zebrafish causes disruptions of heart development. Besides their role as ion-conducting pores, ion channels seem to impinge on cardiogenesis in an ion flux independent manner. However, further studies are required to learn more about the mechanisms how ion channels exert influence on heart development. This paper focuses on the recent findings on the role of ion channels in cardiac development in zebrafish.

## 2. Role of Ion Channels in Heart Development

### 2.1. Sodium Channels

In excitable cells, voltage-gated sodium channels permit the rapid sodium influx and thus are accountable for the initial upstroke of the action potential (phase 0 depolarization). The structure of voltage-gated sodium channels resembles the assembly of calcium channels and other ion channels. Each channel consists of a pore-forming *α* subunit (Na_*v*_ 1) and auxiliary *β* subunits [9]. In chick and mouse it is already known that coordinated electric activity of cardiomyocytes is not required for early heart development [10–12]. Furthermore, results of studies on the zebrafish troponin T mutant *silent heart* showed that these mutants lack both contraction and circulation but cardiogenesis proceeds normally [13]. 

In zebrafish, eight genes encode for *α* subunits of sodium channels: four sets of duplicated genes are termed as scn1Laa & scn1Lab, scn4aa & scn4ab, scn5Laa & scn5Lab, and scn8aa & scn8ab [14–16]. scn5Laa and scn5Lab are phylogenetically related to SCN5A in mammals (Na_*v*_ 1.5) [9, 17]. At 24 hpf scn5Laa mRNA is detected by whole mount in situ hybridisation in regions of the future heart tube [18]. At later stages of embryonic and larval development (at 52 hpf and 104 hpf) scn5Laa and scn5Lab expression is observed in the heart and the nervous system [17]. Transcripts of both genes can be identified by reverse transcriptase polymerase chain reaction assays already in early embryonic stages (0 to 12 hpf) [17]. Morpholino antisense oligonucleotide-mediated knockdown of the cardiac sodium channels scn5Laa or scn5Lab in zebrafish lead to dysmorphic and hypoplastic hearts. scn5Laa morphant hearts show significant defects of both atrial and ventricular chamber morphogenesis and cardiac looping by 58 hpf. During later stages of heart development both chambers remain small. The ventricle displays a single cardiomyocyte layer; trabeculae are missing. Quantification of cardiomyocyte numbers by 62 hpf reveals a significant reduction in comparison to controls. The reason for this reduction of heart cells seems to be a decreased number of differentiating cardiomyocytes at early embryonic stages. Quantification of future heart cells in the cardiac cone at the 22-somite stage, approximately at 20 hpf, reveals significantly reduced numbers compared to wild-type and control-injected embryos. Double knockdown of both cardiac sodium channel genes scn5Laa and scn5Lab leads to an even more severe disruption of cardiac development compared to a single knockdown of either gene. These scn5Laa scn5Lab morphants show even fewer numbers of heart cells. The defects in the heart are not caused by an increase of apoptosis. Thus, these abnormalities seem to be due to early disruption of cardiomyocyte differentiation and proliferation [17].

Injection of either scn5Laa or scn5Lab translation inhibitor Morpholino resulted in a reduction of cardiogenic transcription factors nkx2.5, gata4, and hand2 at the 6-somite stage in the anterior lateral mesoderm. This effect on nkx2.5 expression is dose dependent. Expression of gata5 as a potent positive regulator of nkx2.5 is unaffected. Additionally the expression of the sarcomeric genes cmlc2 and vmhc is decreased at 16-somite stage [17].

When the voltage-gated sodium channel Na_*v*_ 1.5 in zebrafish is blocked either by injection of different sodium channel blockers into the pericardial space of the embryonic heart or by penetration via bathing solution, normal formation of the heart tube is observed. In addition rearing in sodium-free media causes no disruption of cardiogenesis. As expected, at later stages conduction abnormalities are caused by blockage of the voltage-gated sodium channel. Activation of this channel by Anemone toxin II or Veratridine delivered by either bathing solution or injection leads to convulsions, but the cardiac development remains unaffected [17]. These findings indicate that the voltage-gated sodium channel exerts influence on cardiac development independent of ion flux.

In summary, the studies on the voltage-gated sodium channel Na_*v*_ 1.5 in zebrafish suggest that the expression of the channel is essential for heart development. Morpholino knockdown of scna5Laa and scna5Lab causes a decreased number of differentiating cells as well as hypoplastic and dysmorphic hearts. Sodium currents seem to be redundant for this effect. Reduced expression levels of nkx2.5, gata4, and hand2 in scna5Laa and scna5Lab morphants imply an essential role of sodium channels in fate determination and differentiation of cardiac cells.

### 2.2. Calcium Channels

Calcium channels are important for the function of the adult heart. The voltage-gated entry of calcium into cardiomyocytes is permitted by the L-type (for long lasting) and the T-type (for threshold or tiny) calcium channel. The L-type channel is the major route for calcium inward current. Calcium entry via L-type channels (LTCC) is crucial to sustain the characteristically long action potential of cardiomyocytes, conduction, and excitation-contraction coupling [9]. In mammals the T-type channel is predominantly expressed in the sinus node, atrio-ventricular node, and atrial cells where it facilitates automaticity and pace-making activities [9]. Adult zebrafish displays a significant T-type-Ca^2+^ current in both atrial and ventricular cardiomyocytes [19]. Intracellularly, calcium facilitates the contraction mechanism by triggering the release of calcium from the sarcoplasmatic reticulum via ryanodine receptor [9]. Furthermore calcium regulates as a second messenger growth and hypertrophy of cells by less understood mechanisms [20]. In addition the C terminus of the LTCC encodes a transcription factor and autoregulates transcription of the channel [21, 22]. 

LTCC are heteromeric protein complexes usually consisting of a pore-forming *α*1 subunit, a modulatory cytoplasmatic *β* subunit, and an ancillary extracellular *α*2 subunit with a transmembrane *δ* subunit and a function-modifying *γ* subunit in noncardiac cells [23–25].

The ion-conducting pore and the voltage sensor lie within the *α*1 subunit of cardiac-specific isoform *α*1C. The subunit consists of four transmembrane domains each containing of six transmembrane segments. It has been reported that LTCC blockers such as 1,4-dihydropyridine applied to chick embryos cause a reduction of the heart size and thickness of the myocardium [26] whereas targeted mutagenesis of the LTCC *α*1C-subunit did not interfere with embryonic cardiac function and growth in mice [27]. 

In zebrafish the embryonic lethal *island beat* mutant is an excellent model to determine the role of LTCC in cardiac development since its pore-forming *α*1C subunit is defective. In *island beat (isl)* mutant embryos a morphologically normal heart tube is formed in its correct position. Endocardium and myocardium are present. Already at that early stage of heart development, defects become noticeable. The embryos lack the characteristic peristaltic contraction of the heart tube. During cardiac development two chambers are generated in *isl  *mutants. In contrast to wild-type embryos the mutants display a smaller ventricular chamber with single-layer cardiac cells. At 60 hpf appositional addition of cardiomyocytes is absent. Consequently the thickening and thereby growth along the long axis of the heart does not occur. At 72 hpf it becomes clear that the number of cardiomyocytes is reduced in mutants compared to wild type. Apoptosis is not evident in *isl* hearts. The atrium remains morphologically normal throughout the early stages of cardiac development. The atrial cells continue to contract in sporadic, uncoordinated manner resembling atrial fibrillation whereas in ventricular cells contraction and blood flow remain absent. At an ultrastructural level myocardial cells of *isl* mutants appear to be normal [28].

The cytoplasmatic *β* subunit of the calcium channel modulates the electrophysiological properties of the channel and is capable of increasing calcium channel activity when coexpressed with *α*1 subunits [25]. Additionally, the *β* subunit of the LTCC belongs to the group of membrane associated guanylate kinases (MAGUK) which have scaffolding function [29]. *β*2 is the predominantly expressed *β* subunit in the heart. *β*2 null mutant mice die during embryogenesis due to absence of cardiac contraction. Knockout of other calcium channel *β* subunits in mice did not lead to embryonic lethal heart failure [30–32]. The calcium channel *β*4 subunit is expressed in fetal hearts of rats and precedes the expression of the *β*2 subunit [33].

In zebrafish the following *β* subunit genes were identified: cacnb1 for the *β*1 subunit, and for *β*2 cacnb2a and -b, *β*3 cacnb3a and -b and for *β*4 cacnb4a and -b. Similar to the results in mice, in zebrafish cacnb4a is expressed in the embryonic heart at 48–72 hpf. cacnb4b is not expressed in the developing heart tube in zebrafish. At this stage neither cacnb2a nor cacnb2b were detected in cardiac tissue by in situ hybridisation [34]. Interestingly, an antisense oligonucleotide mediated Morpholino knockdown either by translation initiation block or by missplicing of the *β*2.1 subunit causes disruptions of heart development in zebrafish. At 48 hpf the knockdown morphants exhibit defects in chamber shaping, cardiac looping, and contractility. By 72 hpf the heart remains linear and a pericardial edema is present. The atria appear to be dilated whereas the ventricle is collapsed. In *β*2.1 depleted zebrafish specification and differentiation of the cardiomyocyte precursors proceeds without any makeable differences compared to wild type. Nevertheless, in the ventricle of morphants at 48 hpf, reduced numbers of cardiomyocytes were detected, whereas in the atrium equal numbers of cells were counted. A decreased rate of proliferating cardiomyocytes seems to be causative since an increased apoptosis rate was not observed. In addition, the *β*2.1 depleted cardiomyocytes differ from wild-type cells morphologically. While normally the cardiomyocytes undergo a transition from a round to a larger, elongated shape during chamber ballooning, the *β*2.1 knockdown cardiomyocytes remain round. The surface of individual cardiomyocytes at the outer curvature of the heart is markedly reduced. Besides these morphological distinctions, the heart function is compromised in *β*2.1 knockdown morphants. Heart rate, ventricle volume, stroke volume, and cardiac output are decreased at 48 hpf. Moreover upon externally applied mechanical shear stress the *β*2.1 morphant heart tube ruptures since cardiomyocytes express less N-cadherin and lack the characteristic banding of sarcomeric actin. These findings implicate besides its role for proliferation and contractility an important scaffolding function of the *β*2.1 subunit [29].

In conclusion, the *α*1C subunit as well as the *β*2.1 subunit influence heart development in zebrafish. Loss of function of the channel subunits, either by mutation or by Morpholino oligonucleotide antisense mediated knockdown, leads to reduced numbers of cardiomyocytes and disruption of chamber morphogenesis. In *island beat* mutants the appositional growth of the ventricle is disturbed and remains mechanically silent. The *β*2.1 subunit Morpholino knockdown morphants exhibit defects in cardiac looping, contractility, and cell integrity.

### 2.3. Sodium-Calcium-Exchanger

Calcium inward currents drive excitation of excitable cells and contraction of myocytes and cardiomyocytes. In order to prepare for the next contraction, calcium must be extruded from the intracellular cytosol to return to a resting state. Two major routes contribute to this extrusion in myocytes and cardiomyocytes: the sarcoplasmatic reticulum Ca^2+^-ATPase (SERCA2), which sequesters intracellular calcium into sarcoplasmatic reticulum and the Sodium-Calcium-Exchanger (NCX1). This exchanger regulates Ca^2+^ concentration according to the electrochemical gradient and catalyzes the exchange of three extracellular sodium ions for one intracellular calcium ion. The relative contributions of both NCX and SERCA2 to the intracellular calcium concentration change during development and vary by species  [35–38]. Homozygous NCX1-deficient mice showed the absence of a normally beating heart and enhanced apoptosis of cardiomyocytes. A dilated pericardium has been described. The homozygous mice died at approximately day 10 of embryonic development *in utero* [39, 40]. In chick embryos disruption of cardiac development and contraction was observed when treated with a NCX inhibitor [41].

Expression of the cardiac isoform NCX1h in zebrafish manifests first at the 12-somite stage in bilateral cardiac primordia. The expression of NCX1h is restricted to the heart throughout the first 5 days of zebrafish embryonic development [35]. It is prominent in the myocardium of ventricle and atrium and barely noticeable in the outflow tract [42]. Embryonic lethal mutation of the cardiac isoform NCX1h in zebrafish causes atrial arrhythmias and a nearly silent ventricle in the zebrafish mutant* tremblor (tre).* Length and morphology of the heart tube appear to be normal. At later stages of cardiac development an abnormal heart function is observed in homozygous *tre* embryos. These defects appear when the cells of the heart tube differentiate into ventricular and atrial cells. The cardiomyocytes of the heart tube fibrillate, contract arrhythmically and rhythmically. Compared to the wild type the ventricle is markedly smaller. Electron microscopy at 48 hpf reveals sparse sarcomeres with uncoordinated assembly in ventricular cells whereas the atrium of *tre* mutants displays no obvious abnormalities in sarcomere formation. The posterior end of the atrium collapses. At 6 days after fertilization embryonic lethality occurs [35, 42].

Injection of a calcium sensitive dye reveals nearly constant and in relation to the atrium elevated calcium levels of ventricular cells in *tre* mutants suggestive of calcium overload [35].

Early cardiac specification and differentiation seems to be unaffected by disruptions in NCX1h since *tre* mutants display normal expression levels of *vmhc*,* cmlc2*,* tbx5*,* irx1*,* amhc*, and *hand2*. These data imply defects in later cardiac differentiation in *tre* mutants leading to dysmorphogenesis and arrhythmias of both cardiac chambers [35, 42].

### 2.4. **Ca^2+^**-ATPase

The second major route for the extrusion of calcium from the intracellular space is by sequestering calcium into the sarcoplasmatic reticulum via the sarcoplasmatic reticulum Ca^2+^-ATPase2 (SERCA2). The cardiac-specific isoform SERCA2a plays an essential role in excitation-contraction-coupling in the heart. Calcium cations are transported into the sarcoplasmatic reticulum in order to reduce the intracellular cytosolic calcium concentration and to refill the calcium stores of cardiomyocytes [36].

Zebrafish SERCA2 is expressed bilaterally in the cardiac precursors throughout heart development and in skeletal muscle. Knockdown of SERCA2 either by Morpholino injection or by treatment with cyclopiazonic acid, a specific inhibitor of SERCA-activity, resulted in defects of cardiogenesis [43, 44]. The hearts of these embryos fail to expand. Heart looping is absent. In contrast to *tre* mutants, both heart chambers contract continuously without fibrillation. The heart rate is reduced and the chamber contractions are weak. At 6 days after fertilization embryonic lethality occurs [35].

When a calcium sensitive dye is injected into SERCA2 morphants waves of calcium entry are observed correlating with their bradycardic heart rate. Unlike in NCX1h mutants a calcium overload is not detectable in SERCA2 morphants [35].

Thus, both Ca^2+^ extrusion routes, NCX1h and SERCA2, are each important for zebrafish heart formation and function, but their loss of function phenotypes are distinct.

### 2.5. Na^+^K^+^-ATPase

Na^+^K^+^-ATPase obtains a crucial role for the establishment of a proper electrochemical gradient across the plasma membrane. Sodium and potassium are pumped across the cell boundaries, ATP is utilized. Both, the cation and ATP-binding sites are located in the *α* subunit and are essential for the catalytic and transport function of the integral membrane protein. Dimerization of *α* and *β* subunit is required for enzyme activity (for review see [52]).

In mammals it has been demonstrated that four isoformes of the *α* subunit of Na^+^K^+^-ATPases and three isoformes of the *β* subunit exist, each showing distinct expression patterns and different affinities to glycosides [52]. Cardiac function is modified by Na^+^K^+^-ATPases through interaction with the Sodium-Calcium-Exchanger NCX. Blockade of Na^+^K^+^-ATPases leads to an increase of Na^+^ concentration in cardiomyocytes. Consequently NCX is inhibited, the intracellular calcium concentration rises and thereby the contractility of cardiomyocytes is enhanced [53, 54].

Studies on mice demonstrated that Na^+^K^+^-ATPase activity is crucial for embryogenesis. *α*1 homozygous knockout mice are embryonic lethal and heterozygous mice have hypocontractile heart beats; *α*2 homozygous knockout mice die during the first day after birth and heterozygous mice show hypercontractility of the myocardium that correlates with the elevated calcium level in cardiomyocytes [55]. Pharmacological inhibition of Na^+^K^+^-ATPase by Ouabain in chick embryos leads to disruptions of early heart development [56]. 

In zebrafish eight isoformes of the *α* subunit and five *β* subunits exist. Three Na^+^K^+^-ATPase isoformes are expressed in the developing heart: *α*1B1 (also known as *α*1a1), *α*2, and *β*1a [57]. 

*Heart and mind (had)* mutants carry defects in Na^+^K^+^-ATPase *α*1B1 isoform. *had* embryos develop a curved body and severe abnormalities in brain and heart. Circulation is not established and the embryos die five days after fertilization [45]. By 21 hpf bilateral cardiac primordia fuse at midline and a shallow heart cone becomes evident. At 24 hpf the *had* mutant's heart remains as a shallow heart cone, whereas in wild type at the same stage development of the primitive heart tube is nearly finished. The heart usually starts beating in the stage of the primitive heart tube. *had* mutants reveal a small heart without contraction after 28 hpf. By 48 hpf both cardiac chambers and both cardiac cell types, endocardium and myocardium, exist. However the heart remains small, the ventricle thin, and the myocardial-endocardial distance is enlarged in Na^+^K^+^-ATPase *α*1B1 defective zebrafish [45]. The expression patterns of the early cardiac development genes *vmhc*, *nkx2.5*, and *cmlc2* are normal in these mutants at approximately 24 hpf, suggesting that *had* is not required for the determination of early cardiac cell fate. After 48 hpf *vmhc* and *irx1* become specific for the ventricle in wild-type zebrafish, whereas *versican* expression usually is localized between atrium and ventricle. In *had* mutants a significant reduction of *irx1* can be observed, whereas *vmhc* is evident in the atrium as well as in the ventricle. *Versican* spreads throughout the whole heart, suggesting a disruption of cardiac chamber specific differentiation [45].

Quantification of cardiomyocytes by counting transgenic green-fluorescent-protein- (GFP-) positive cells reveals similar numbers in both wild-type and *had* mutant. The primitive heart is markedly shorter and thereby the GFP signal more intense. Hence, extension of the heart tube seems to be disrupted in *had* mutants [45].

As mentioned above, the heart of *had* mutants does not beat at 24 hpf. After 48 hours of development a bradycardic heart rhythm is established, and cardiac contractility is diminished. Treatment with Ouabain, a Na^+^K^+^-ATPase inhibitor, and injection of a Morpholino antisense oligonucleotide to wild-type zebrafish phenocopies *had* mutants [45, 56].

The *α*2 subunit of Na^+^K^+^-ATPase is also expressed in the developing heart of zebrafish. By injection of a Morpholino antisense oligonucleotide targeting the translation initiation site of this isoform a distinct phenotype occurs. The proper cardiac laterality is perturbed; whereas in wild-type embryos the hearts shifts to the left side by 24 hpf (*cardiac jogging*), the heart of *α*2 knockdowns remains in midline or is displaced to the right side in 51% of all injected embryos. Cardiac looping is abnormal in half of the knockdown morphants [45].

In summary in zebrafish both Na^+^K^+^-ATPase isoformes *α*1B1 and *α*2 contribute to cardiac development in zebrafish in different ways: *α*1B1 defective mutants exhibit abnormalities in heart tube extension and ventricular cardiomyocyte differentiation as well as bradycardia and hypocontractility. *α*2 isoform contributes to cardiac laterality [45]. 

### 2.6. Potassium Channels

Cardiac repolarization depends mainly upon outward currents of potassium cations. Different potassium channels are involved in the repolarization process. The outward current is provided by three delayed rectifier K^+^-currents in humans (rapidly activating I_kr_, slow I_ks_, and ultrarapid I_kur_). Inward currents are provided by cardiac inward rectifier potassium channels encoded by the Kir superfamily [9, 46].

KCNH2 or hERG (human ether-á-go-go related gene) encodes the *α* subunit underlying rapidly activating delayed rectifier K^+^-current I_kr_  [9]. In zebrafish hearts a highly expressed orthologue has been found: zERG. Both hERG and zERG contain six transmembrane segments, one Per-Arnt-Sim (PAS) domain and a cyclic-nucleotide-binding region. An overall amino acid identity between the two species of 59% is shared, whereas in certain domains such as the transmembrane and the catalytic domains, a markedly higher homology is observed [47, 58]. Both ERG channels, hERG and zERG, feature similar biophysical properties [48]. Several arrhythmias are linked to mutations in zERG channels including Short-QT-Syndrome and Long-QT-Syndrome [47, 49].

A gain of function mutation in the homozygous zebrafish mutant* reggae (reg)* prevents ERG channel inactivation. This recessive mutation resides within the voltage sensor of zERG. As a consequence accelerated repolarization, shortened action potential duration, and premature channel reactivation occur. *Reg* mutants display intermittent cardiac arrest, sinuatrial block, and atrial fibrillation already at embryonic stages. In adulthood the zebrafish demonstrates shortened QT-intervals on surface electrocardiograms. Besides the arrhythmias, no disruptions in cardiac development are observed. Essential steps of zebrafish heart development proceed properly in homozygous *reg* mutant embryos without any abnormalities [47, 50].

The zebrafish mutant *breakdance (bre)* is the first animal model for Long-QT-Syndrome. Ventricular bradycardia and 2 : 1 block between atrium and ventricle occur. Causative is a loss of function mutation in the PAS-domain of zERG which leads to an impairment of ERG protein trafficking and hence reduced expression of zERG at the cell membrane. Cardiac repolarization and thereby the QT-interval is prolonged. Besides arrhythmias the *bre*  mutants exhibit no specific phenotype. Cardiac development appears to be normal [49, 58]. Another zebrafish model for Long-QT-Syndrome is provided by mutants with defects residing in KCNH2, respectively, zERG. Two different loss of function mutations (S290^−/−^, S213^−/−^) cause a mechanically silent ventricle and pericardial edema. By 33 hpf the ventricle does not contract and appears to be collapsed. Atrial function and morphology seem to be unaffected in these mutants at this developmental stage. At the 48 hpf stage pericardial edema is noticeable and increases gradually. After 10 days after fertilization the homozygous zebrafish mutants die [51].

Neither Morpholino antisense oligonucleotide translation block of zERG nor incubation with zERG inhibitor E-4031 in bathing solution cause disruptions in cardiac development whereas arrhythmias, particularly bradycardia and irregular atrial rhythms, can be provoked [17].

In summary, the different mutations of zERG cause no specific cardiac phenotype. The regular heart rhythm is disturbed and arrhythmias occur, whereas no major structural alterations appear in the developing heart.

## 3. Conclusion

Several studies on chick, mouse, zebrafish, and other species highlight the impact of ion channels on heart development. Owing to the advantage of survival without circulation, the zebrafish facilitates research on these cardiovascular defects without distortion by hypoxia and opens up new possibilities. Various methods enforced the results of studies on mammals and extended the previous knowledge: ion channel mutants, knockdown by Morpholino antisense oligonucleotides, and pharmacological modulation exhibit perturbed heart development at different time points (Table 1).

Voltage-gated sodium channels impinge on early cardiogenic differentiation, whereas the Sodium-Calcium-Exchanger NCX1h influences later stages of the differentiation. Heart looping hinges on proper function of Ca^2+^-ATPase SERCA2, while the voltage-gated L-type calcium channel affects ventricular morphogenesis. The Na^+^K^+^-ATPase isoformes have distinct functions: *α*1B1 facilitates cardiac differentiation and extension of the heart tube. *α*2 isoform modifies laterality of cardiac precursors. The impact of the variety of potassium channels is least understood. Mutations in zERG cause no major structural alterations during heart development.

The zebrafish's heart proved to be a valuable model for congenital and acquired cardiovascular disease in humans. The findings on the role of ion channels in zebrafish embryos provide new insights into cardiac development. In addition to human pedigree studies and candidate gene screens the zebrafish model might help to understand the underlying mechanisms leading to congenital heart disease in humans. As a consequence the invention of causative treatment targeting the ion channels might become possible.

However the heart begins to function during its formation. Cardiogenic differentiation and morphogenesis occur simultaneously and interact mutually. Some of the developmental steps of the heart seem to be independent of proper cardiomyocyte function while others depend on normal function. The recent findings on ion channels and heart development highlight the crucial role of ion channels for specification, differentiation, and morphogenesis. Yet, the mechanisms how the ion conducting pores exert influence on heart development remain unclear. Ion flux dependent and independent functions of ion channels in the developing heart seem to exist. Hence there is still a lot to be learned from zebrafish.

## Figures and Tables

**Table 1:** Affected ion channel and effect on heart development.

Affected ion channel	Zebrafish phenotype and effect on heart development	Reference
Na_*v*_ 1.5 (scn5Laa, scn5Lab)	(i) Morpholino knockdown: hypoplastic, dysmorphic heart, reduced numbers of cardiomyocytes (ii) pharmacological modification: normal heart tube formation	[17]

LTCC *α*1C subunit	(i) *Island beat (isl): *loss of function; hypoplastic and silent ventricle, reduced numbers of ventricular cardiomyocytes; atrial fibrillation	[28]

LTCC *β*2.1 subunit	(i) Morpholino knockdown: defects in cardiac looping and ballooning, reduced numbers and proliferation of cardiomyocytes, disrupted cell integrity; bradycardiac and weakly contractile heart rhythm (ii) pharmacological modification with nifedipin resembles the morphants' phenotype	[29]

NCX1h	(i) *Tremblor (tre):* loss of function; hypoplastic and nearly silent ventricle, disruptions of sarcomere assembly in the ventricle; atrial fibrillation	[35]

SERCA2a	(i) Morpholino knockdown, pharmacological modification: absent cardiac looping, no expanding of cardiac chambers, bradycardia	[35]

Na^+^K^+^-ATPase *α*1B1 subunit	(i) *heart and mind (had): *loss of function, disturbed heart tube elongation and cardiogenic differentiation	[45]

Na^+^K^+^-ATPase *α*2 subunit	(i) Morpholino knockdown: perturbed cardiac looping and laterality	[45]

zERG	(i) *reggae (reg): *gain of function; Short-QT-Syndrome, no effect on heart development (ii) *breakdance (bre): *loss of function; Long-QT-Syndrome, no effect on heart development (iii) S290^−/−^, S213^−/−^: loss of function; Long-QT-Syndrome (iv) Morpholino knockdown, pharmacological modification: no effects on heart development	[46–51]
